# Tardive Dyskinesia, Oral Parafunction, and Implant-Supported Rehabilitation

**DOI:** 10.1155/2016/7167452

**Published:** 2016-12-06

**Authors:** S. Lumetti, G. Ghiacci, G. M. Macaluso, M. Amore, C. Galli, E. Calciolari, E. Manfredi

**Affiliations:** ^1^Centro Universitario di Odontoiatria, SBiBiT, Università degli Studi di Parma, Parma, Italy; ^2^Sezione di Psichiatria, Dipartimento di Neuroscienze, Riabilitazione, Oftalmologia, Genetica e Scienze Materno-Infantili, Università degli Studi di Genova, Genova, Italy; ^3^Centre for Oral Clinical Research, Queen Mary University of London, London, UK

## Abstract

Oral movement disorders may lead to prosthesis and implant failure due to excessive loading. We report on an edentulous patient suffering from drug-induced tardive dyskinesia (TD) and oral parafunction (OP) rehabilitated with implant-supported screw-retained prostheses. The frequency and intensity of the movements were high, and no pharmacological intervention was possible. Moreover, the patient refused night-time splint therapy. A series of implant and prosthetic failures were experienced. Implant failures were all in the maxilla and stopped when a rigid titanium structure was placed to connect implants. Ad hoc designed studies are desirable to elucidate the mutual influence between oral movement disorders and implant-supported rehabilitation.

## 1. Introduction

Tardive dyskinesia (TD) is characterised by involuntary, repetitive, and purposeless movements, which may involve chewing motions, cheek puffing, tongue protrusion, and lip pursing. Movements of other body segments may also occur, and symptoms may appear during sleep and/or wakefulness [1, 2]. Most often TD represents a side effect of antipsychotic medications [3, 4]. Typical and, at a lower rate, atypical antipsychotics may induce TD probably by increasing dopamine sensitivity in the nigrostriatal pathway, especially for D2 dopamine receptor [5–9]. Other drugs, as antiemetic metoclopramide and antidepressants, have been linked to TD, although with much lower frequency [10–13]. It is important to underline the fact that these drugs are capable of inducing diverse movement disorders, as dystonia [14], myoclonus [15], “rabbit syndrome” [16], and sleep bruxism [17]. The latter has been linked particularly to selective serotonin reuptake inhibitors (SSRIs) [18, 19].

The term “tardive” was originally used to indicate the most frequent timing of dyskinesia onset, after at least 3 months of therapy. However, the appearance of dyskinetic symptoms is not dose-related and may occur either after a short or a long time of drug use, and it is generally accepted that most patients will eventually fall ill with the disorder if they remain on neuroleptics long enough.

Oral parafunctions (OP) include many activities occurring during the awake state, the commonest being prolonged steady mandibular postures and jaw clenching [20]. They can be classified in primary, or idiopathic, and secondary, when they originate from a neurological or psychiatric disease or represent a side effect of a medication or a recreational drug. Alcohol intake and cigarette smoking may also contribute [21]. They have been associated with psychiatric disorders as well as psychosocial factors like stress and anxiety [22–24].

TD may have dental implications, as it causes attritions and abfractions on natural teeth [25, 26]. It also represents a risk factor for the prosthetic management of the patient, worsening the stability of complete dentures and increasing the risk of prostheses breaks. Additionally, TD-provoking drugs can induce changes of salivary flow, which worsen patient's adaptation to removable prostheses [27–30]. Implant-supported fixed rehabilitation may appear as a valuable therapeutic option, as it improves prostheses stability and has positive psychosocial effects [31, 32]. However, oral movement disorders may cause excessive load of the prostheses, which in turn may affect implant outcome [33, 34], and jeopardise simple or complex rehabilitative procedures and tardive dyskinesia represents a particularly critical situation for implant rehabilitation [35, 36].

We here report a case of implant-supported fixed rehabilitation in an edentulous patient with extreme loading conditions due to TD and OP.

## 2. Case Presentation

A 58-year-old Caucasian man complaining of unsatisfactory removable prostheses was admitted to the dental clinic. Remote anamnesis revealed history of alcohol abuse associated with impulsive behaviour, with the start of medical therapies dating back to 2004. The patient was at that time suffering from major depression and narcissistic personality disorder and was administered a multiple pharmacotherapy. He was treated with Citalopram 40 mg/day, aimed at controlling depression, from 2004 to 2007; an occasional treatment with Paroxetine 30 mg/day was performed in 2004 for 90 days. During the same year, the patient took Promethazine 25 mg/day. Valproic acid 1 g/day and Oxcarbazepine 1.2 g/day were prescribed up to now as anticonvulsants. The patient was also administered benzodiazepines: Lorazepam 2.5 mg/day from 2004 to 2006, Triazolam 0.25 mg/day, and Diazepam 2 mg/day from 2005 to 2006. A temporary treatment with the second-generation antipsychotic Olanzapine 5 mg/day was carried out for 90 days in 2005. The patient also assumed Trazodone 75 mg/day. In 2007, the antidepressant Venlafaxine substituted Citalopram, with doses increasing to the current posology of 150 mg/day. Clonazepam 5 mg/day was administered since 2007, substituting previously used benzodiazepines. In 2008, the patient received Hydroxyzine 25 mg/day. During 2011, Zolpidem 4 mg/day was prescribed to the patient. Olanzapine 5 mg/day was permanently reintroduced into the therapy in April 2012.

The patient has been suffering from TD as a side effect of drugs since 2009. The involuntary movements he presented were repetitive stereotyped chewing motions and lip protruding. The movements were probably present also during sleep, but the patient did not accept further ambulatory sleep study.

OP was also evident, as prolonged steady mandibular postures and habit of teeth clenching: these activities, differently from the repetitive movements of TD, could be voluntarily stopped and were exacerbated in state of psychological anxiety.

The patient had been smoking 20 cigarettes/day since many years.

**Figure 1:**
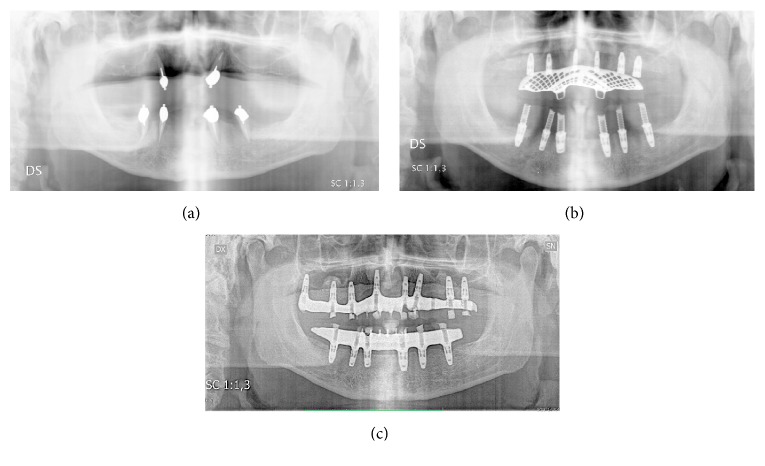
Orthopantomography of the patient at first visit (a), 18 weeks (b), and 96 weeks (c).

Clinical examination of the oral cavity revealed the presence of 2 residual teeth in the maxilla (canines) and 4 residual teeth in the mandible (2 canines and 2 premolars), restored with post and ball attachment to, respectively, stabilise maxillary and mandibular overdentures. All teeth were affected by severe reduction of periodontal support and showed class III mobility. Marked gingival inflammation was also evident. Orthopantomography attested horizontal bone loss around the remaining teeth and generalised vertical atrophy of the alveolar processes (Figure 1(a)).

Treatment consisted of extraction of the remaining hopeless teeth (time: T0) and subsequent full mouth rehabilitation with implant-supported fixed prostheses. After thorough discussion, the patient approved this therapeutic option.

Twelve weeks following T0, a CT scan (Siemens Somatom Emotion 6; Siemens, Erlangen, Germany) was performed to plan implant insertion (NobelGuide planning software; Nobel Biocare, Göteborg, Sweden). Two weeks later, the patient underwent bilateral sinus lift with lateral window approach. Autologous bone chips plus deproteinised bovine bone granules (Bio-Oss; Geistlich Pharma AG, Wolhusen, Switzerland) were used as grafting materials. In the course of the same surgical intervention, six dental implants (NobelReplace, Nobel Biocare, Göteborg, Sweden, as all the remaining implants employed) were placed in the maxilla with an insertion torque > 30 N/cm. The patient was provided with a provisional complete denture to wear during the planned 24-week healing period.

The mandible was rehabilitated 18 weeks after T0 by means of 6 dental implants inserted with a torque > 40 N/cm and immediately restored with a provisional screw-retained acrylic bridge. The implants were all 10 mm long and 4.3 diameter, except the 4.6 implant, which had a 5 mm diameter (Figure 1(b)). A group function occlusal scheme was created, with accurate control of contacts in intercuspal position and lateral and protrusive movements. This occlusal scheme was maintained in both provisional and final prostheses.

At 32 weeks, a complete mandibular implant-supported screw-retained prosthesis was finalised with composite teeth and milled titanium framework.

At 36 weeks, the implant in 1.1 position failed. At a routine control visit, the implant was exposed and mobile, with no symptoms. It was therefore removed and substituted with a larger implant. At the same time point, the remaining maxillary implants were loaded with a provisional implant-supported screw-retained acrylic prosthesis.

Four weeks later (at 40-week time point), the implants in 2.2 and 2.6 positions failed, with no apparent sign of infection. The implant in 2.2 site was replaced with a larger one. This was not possible for the 2.6 implant; thus an implant was inserted more distally, in 2.7 position. An additional implant was placed in 1.7. A provisional acrylic bridge was then placed, using all the maxillary implants.

At 44 weeks, the implant in 2.4 site showed severe marginal bone loss on the vestibular side, which was managed by means of a deproteinised bovine bone granular graft (Bio-Oss; Geistlich Pharma AG, Wolhusen, Switzerland). Despite this intervention, the implant failed at 48 weeks and was replaced with a longer implant, inserted apically. Another implant was positioned in the adjacent 2.3 area.

At the following monthly follow-up visits, the provisional maxillary bridge frequently showed cracks, which underwent mostly unnoticed by the patient. All cracks could be repaired.

At 60 weeks, the maxillary acrylic bridge broke into two pieces, and implants 2.4 and 2.7 were removed, due to their loss of osseointegration in the absence of infection signs. Implants were placed in 2.6 and 2.7 sites. A new acrylic full arch bridge was screwed to all the maxillary implants.

**Figure 2:**
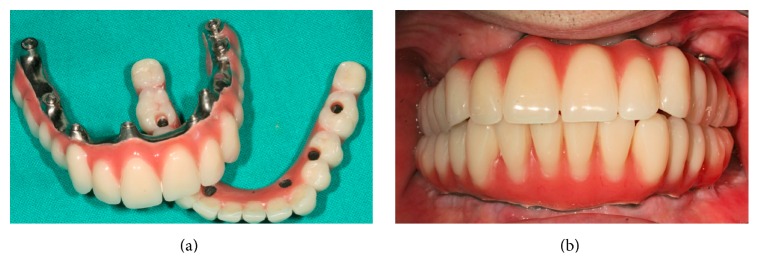
Final prostheses delivered to the patient at 96 weeks.

**Figure 3:**
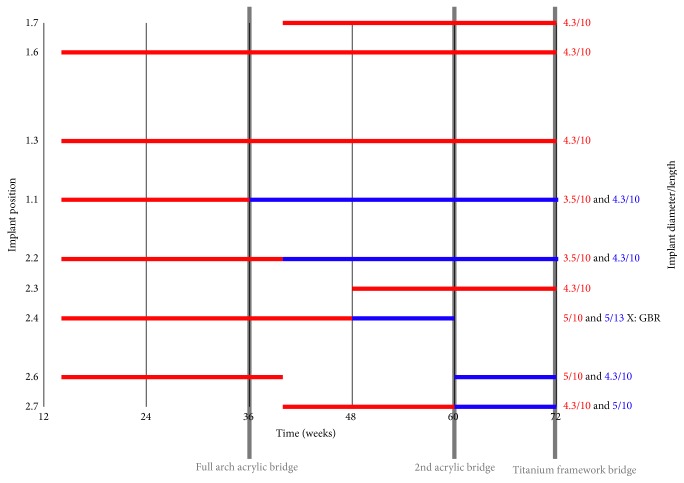
Time course of maxillary implant insertions and failures, ordered by position. In red a given implant first positioning and in blue the substituting implant. The initial planning included 6 implants with delayed loading. Five implants failed. The maxilla was finally rehabilitated at 72 weeks with a screwed titanium milled framework with acrylic composite teeth supported by 8 implants. No additional implant failures occurred.

At 72 weeks, the patient was provided with a maxillary screw-retained prosthesis with composite teeth and milled titanium framework (Figures 2(a) and 2(b)). From this moment to the 96-week follow-up visit, no further maxillary implant was lost and the prosthesis performed satisfactorily. A time course of maxillary implant insertions and failures is described in Figure 3.

At 96 weeks, all mandibular implants were successful (Figure 1(c)), as they had good stability and showed no marginal bone loss, no inflammation, nor any symptoms. The mandibular full arch bridge performed adequately during the whole examination period, showing some sign of attrition.

TD appeared as a permanent condition during the observation period. It was not possible to prescribe a pharmacological therapy specifically aimed at controlling TD due to the underlying psychiatric disease of the patient. The patient refused night splint therapy.

Massive efforts to teach self-control of OP were undertaken, but the effect was highly variable in time and, overall, poor.

## 3. Discussion

Oral parafunctions may alter occlusal loads of natural teeth, prostheses, and dental implants, both in terms of force direction and intensity. Dental attrition, abfraction, and occlusal pits on natural teeth have been documented in patients with self-reported parafunctions. Many authors suggested that oral parafunctions represent a risk factor for marginal bone loss around implants and implant failure [37–40].

TD and other oral movement disorders can similarly generate increased tooth or implant loads. A case of traumatic granulomatous tissue at the implant site of a patient with TD has been reported [41]. Other studies showed successful results of implant-retained overdentures in patients with neurological movement disorders involving oral and perioral areas [35, 36, 42–46].

As a matter of fact, the influence of increased load on implants still represents a matter of debate and no clear-cut conclusion can be drawn from the published literature [47–54]. This is in part due to the inherent difficulty in quantifying the load transferred to implants in a clinical setting. In the present case, all implant failures occurred in absence of infection. They were likely due to the extreme mechanical loads caused by coexisting severe TD and OP.

The different survival rate of maxillary versus mandibular implants (57% versus 100%) may be related to different factors. The anatomical characteristics of the jaws arguably play a role, the mandible being mainly composed of cortical compact bone. The literature has consistently reported high implant success rates in the mandible, also employing immediate loading protocols. Maxillary bone is generally less favourable, due to its lower density and higher amount of trabecular bone [55, 56], which may hinder the rehabilitation techniques that are applied [57, 58]. Moreover, sinus lift interventions and grafted materials used may play an influence on implant outcome [59, 60]. In our case, implants placed in areas of sinus lifts were successful in 1.6 and 1.7 positions, while they failed on the opposite side in 2.6 and 2.7 positions.

Prosthetic aspects are of the utmost importance in determining the load transfer to implants. Unfortunately, as of today, there is no clear evidence-based concept of “ideal occlusal scheme” to apply in implant-supported prosthodontics [61, 62]. A review assessed the importance of decreasing cuspal interferences, centralising forces along implant axes and avoiding cantilevers [63]. Klineberg recommended an occlusal design with narrow occlusal table, with central fossa loading in intercuspal contact and low cusp inclination [64, 65]. Occlusal canine guidance instead of group function in patients with OP has also been proposed [66].

Prosthetic breaks represent one of the commonest complications in the rehabilitation of patients suffering from oral parafunctions: it has been reported that ceramic crowns have high risk of fracture, especially when they articulate one with the other [67, 68]. Skalak first hypothesised that resilient prosthetic materials help reducing overloads and so recommended the use of acrylic resin teeth in implant-supported prostheses [69]. Others proposed to use a metal occlusal surface, which combine resiliency and resistance properties [70].

In this case, the patient frequently broke the unreinforced acrylic resin maxillary full arch prosthesis. Such loss of integrity may have represented a further cause of mechanical stress build-up at certain implant areas, together with the inherent flexibility of this type of prosthesis. We believe that the latter characteristic was of the utmost importance, since implant failure stopped after installing a prostheses with rigid titanium milled framework. This was probably due to a better load distribution among implants. It is also possible that specific implant surfaces may prove better suitability to withstand abnormal mechanical loads [71], and this aspect should be further explored. This could be consistent with a previous report by Amornvit et al. showing a successful implant rehabilitation with a balanced occlusal scheme, which may have compensated the excessive occlusal loading [36]. The patient's personal history as a heavy smoker should also be considered, as smoke has been shown to be related to risk for and severity of bruxism [72, 73] and may have affected the severity of the symptoms, but may also have impacted on implant survival as smoke has been associated with increased implant failure and complications [74–77].

We conclude that abnormal occlusal loads related to TD and OP played a key role in determining repeated prosthetic breaks and implant failures in the present case. The use of a bridge with a rigid framework stopped implants failures. Longitudinal controlled studies to investigate implant-supported rehabilitation in TD and other extreme load-generating movement disorders are needed.
